# Supplementary material for: Prognostic factors for outcomes of idiopathic sudden sensorineural hearing loss: protocol for the SeaSHeL national prospective cohort study
Source: BMJ Open. 2020 Sep 28;10(9):e038552. doi: 10.1136/bmjopen-2020-038552 (PMC7523222; doi:10.1136/bmjopen-2020-038552)

BMJ Open is committed to open peer review. As part of this commitment we make the peer review history of every article we publish publicly available.

When an article is published we post the peer reviewers' comments and the authors' responses online. We also post the versions of the paper that were used during peer review. These are the versions that the peer review comments apply to.

The versions of the paper that follow are the versions that were submitted during the peer review process. They are not the versions of record or the final published versions. They should not be cited or distributed as the published version of this manuscript.

BMJ Open is an open access journal and the full, final, typeset and author-corrected version of record of the manuscript is available on our site with no access controls, subscription charges or pay-per-view fees (http://bmjopen.bmj.com).

If you have any questions on BMJ Open's open peer review process please email info.bmjopen@bmj.com

BMJ Open

Prognostic factors for outcomes of idiopathic Sudden Sensorineural Hearing Loss: protocol for the SeaSHeL national prospective cohort study

Journal:	*BMJ Open*
Manuscript ID	bmjopen-2020-038552
Article Type:	Protocol
Date Submitted by the Author:	14-Mar-2020
Complete List of Authors:	Mandavia, Rishi; National Institute for Health Research, University College London Hospitals Biomedical Research Centre; University College London Ear Institute, evidENT<br>Hannink, Gerjon; Radboudumc, Department of Operating Rooms<br>Ahmed, Muhammad ; University College London Ear Institute, evidENT; University of Leeds School of Medicine<br>Premakumar, Yaami; Chelsea and Westminster Hospital<br>Chu, Timothy; 4. SFO UK Students and Foundation Doctors in Otolaryngology; Newcastle University School of Clinical Medical Sciences<br>Blackshaw, Helen; National Institute for Health Research, University College London Hospitals Biomedical Research Centre; University College London Ear Institute, evidENT<br>Ferdous, Tanjinah; National Institute for Health Research, University College London Hospitals Biomedical Research Centre; University College London Ear Institute, evidENT<br>Mehta, Nishchay; National Institute for Health Research, University College London Hospitals Biomedical Research Centre; University College London Ear Institute, evidENT<br>Manjaly, Joseph; National Institute for Health Research, University College London Hospitals Biomedical Research Centre; University College London Ear Institute, evidENT<br>Khan, M. M.; INTEGRATE The National ENT Trainee Research Collaborative; SFO UK Students and Foundation Doctors in Otolaryngology<br>Schilder, Anne; National Institute for Health Research, University College London Hospitals Biomedical Research Centre; University College London Ear Institute, evidENT
Keywords:	OTOLARYNGOLOGY, Adult otolaryngology < OTOLARYNGOLOGY, Audiology < OTOLARYNGOLOGY

SCHOLARONE™
Manuscripts

I, the Submitting Author has the right to grant and does grant on behalf of all authors of the Work (as defined in the below author licence), an exclusive licence and/or a non-exclusive licence for contributions from authors who are: i) UK Crown employees; ii) where BMJ has agreed a CC-BY licence shall apply, and/or iii) in accordance with the terms applicable for US Federal Government officers or employees acting as part of their official duties; on a worldwide, perpetual, irrevocable, royalty-free basis to BMJ Publishing Group Ltd ("BMJ") its licensees and where the relevant Journal is co-owned by BMJ to the co-owners of the Journal, to publish the Work in this journal and any other BMJ products and to exploit all rights, as set out in our licence.

The Submitting Author accepts and understands that any supply made under these terms is made by BMJ to the Submitting Author unless you are acting as an employee on behalf of your employer or a postgraduate student of an affiliated institution which is paying any applicable article publishing charge ("APC") for Open Access articles. Where the Submitting Author wishes to make the Work available on an Open Access basis (and intends to pay the relevant APC), the terms of reuse of such Open Access shall be governed by a Creative Commons licence – details of these licences and which Creative Commons licence will apply to this Work are set out in our licence referred to above.

Other than as permitted in any relevant BMJ Author's Self Archiving Policies, I confirm this Work has not been accepted for publication elsewhere, is not being considered for publication elsewhere and does not duplicate material already published. I confirm all authors consent to publication of this Work and authorise the granting of this licence.

For peer review only

Prognostic factors for outcomes of idiopathic Sudden Sensorineural Hearing Loss: protocol for the SeaSHeL national prospective cohort study

Rishi Mandavia[1,2,3,4,5] (r.mandavia@ucl.ac.uk),

Gerjon Hannink[6] (gerjon.hannink@radboudumc.nl),

Muhammad N Ahmed[2,10] (um17mna@leeds.ac.uk),

Yaamini Premakumar[7] (yaami.p@gmail.com),

Timothy Shun Man Chu[4,8] (t.chu1@newcastle.ac.uk),

Helen Blackshaw[1,2] (h.blackshaw@ucl.ac.uk),

Tanjinah Ferdous[1,2,5] (tanjinah.ferdous@nhs.net),

Nischay Mehta[1,2,3,5] (nishchay.mehta.12@alumni.ucl.ac.uk),

Joseph Manjaly[1,2,5] (joseph.manjaly@nhs.net),

Maha Khan[3,4,9] (maha.khan@doctors.org.uk),

Anne G M Schilder[1,2,5] (a.schilder@ucl.ac.uk)

On behalf of the SeaSHeL Collaborative

1. National Institute for Health Research, University College London Hospitals Biomedical Research Centre, 90 Tottenham Court Road, W1T 4TJ, United Kingdom
2. evidENT, Ear Institute, University College London, 90 Tottenham Court Road, W1T 4TJ, United Kingdom
3. INTEGRATE The National ENT Trainee Research Collaborative, United Kingdom
4. SFO UK Students and Foundation Doctors in Otolaryngology, United Kingdom
5. Royal National ENT and Eastman Dental Hospitals, University College London Hospitals Trust, London, United Kingdom

For peer review only

6. Radboud University Medical Centre, Nijmegen, Netherlands

7. Chelsea and Westminster Hospital, London, United Kingdom

8. Newcastle University Medical School, United Kingdom

9. Health Education North West, United Kingdom

10. University of Leeds School of Medicine, United Kingdom

Corresponding author.

Dr Rishi Mandavia

Address: NIHR UCLH BRC and evidENT, Ear Institute, University College London, 90 Tottenham Court Road, London W1T 4TJ

Email: r.mandavia@ucl.ac.uk

Telephone: +44 (0) 20 3108 9327

Word Count: 1792

Keywords

Sudden Sensorineural Hearing Loss, Prospective cohort study, Quality of life, Prognostic model, Steroids

For peer review only

ABSTRACT:

Introduction

The mainstay of treatment for idiopathic sudden sensorineural hearing loss (SSNHL) includes oral steroids, intratympanic steroid injections or a combination of both. NICE, in their recent hearing loss guidelines, highlighted the paucity of evidence assessing the comparative effectiveness of these treatments; and the NIHR HTA Programme has since released a commissioned call for a trial to identify the most effective route of administration of steroids as a first-line treatment for idiopathic SSNHL. For such trials to be run effectively, reliable information is needed on patients with SSNHL: where they present, numbers, demographics, treatment pathways, as well as outcomes. This study will collect these data in a nationwide cohort study of patients presenting with SSNHL across 97 NHS Trusts. The study will be delivered through ENT trainee networks, the NIHR Clinical Research Network (CRN) Audiology Champions, and the NIHR CRN. Importantly, this study will also provide a dataset to develop a prognostic model to predict recovery for patients with SSNHL.

Objectives

1. To map the patient pathway and identify the characteristics of adult patients presenting to NHS ENT and hearing services with SSNHL.

2. To develop a prognostic model to predict recovery for patients with idiopathic SSNHL.

3. Establish the impact of idiopathic SSNHL on patients' quality of life (QoL)

Methods and analysis

-Study design: National multicentre prospective cohort study across 97 NHS Trusts.

For peer review only

-Inclusion criteria: Adult patients presenting to NHS ENT and hearing services with SSNHL.

-Outcomes: Change in auditory function; change in QoL score.

-Analysis: Multivariable prognostic model, using pre-specified candidate predictors. Mean change in QoL scores will be calculated from initial presentation to follow up.

Ethics and dissemination

Health Research Authority (HRA) and NHS Research Ethics Committee (REC) approved the study. Publication will be on behalf of all study sites and collaborators.

Registration details

Clinicaltrials.gov NCT04108598

ARTICLE SUMMARY

Strengths and limitations of this study

- Taking place across 97 NHS Trusts, the SeaSHeL study represents the first nationwide prospective cohort study of adults presenting to ENT and hearing services with SSNHL.
- The study will provide key data to inform the design and delivery of an NIHR HTA Programme commissioned call for a trial to identify the most effective route of administration of steroids as a first-line treatment for idiopathic SSNHL.
- It is uniquely delivered by INTEGRATE (National ENT Trainee Research Network), SFO-UK (Student and Foundation Doctors in Otolaryngology), NIHR Clinical Research Network (CRN) Audiology Champions, and the NIHR CRN.

For peer review only

- The study will provide a unique dataset to develop a prognostic model to predict recovery for patients with SSNHL, as well as identify the impact of the condition on patient quality of life.

INTRODUCTION

Each year approximately 20 people per 100,000 experience a sudden hearing loss in one ear that is located in the inner ear.[1] This is called sudden sensorineural hearing loss, SSNHL, defined as a rapid loss of hearing occurring over three days, of 30 dB (decibel) or more, over at least 3 contiguous sound frequencies.[2] SSNHL is predominantly unilateral and the degree of hearing loss can range from mild to profound. In 71% to 90% of cases, the cause is unknown despite investigation, and these cases are termed idiopathic SSNHL.[3,4]

Idiopathic SSNHL is a serious condition, adversely impacting people's lives[5] with research showing associations with emotional distress, depression, difficulties at work and impaired social integration.[6,7] Its prognosis is poorly understood and may depend on age, comorbidities, degree of hearing loss, audiometric configuration, presence of vertigo, treatment received, time between onset of hearing loss and treatment and other factors. It is estimated that 32% to 65% of cases of idiopathic SSNHL recover spontaneously, although clinical experience suggests that this may be an overestimation, with further research required in this area.[2,8,9]

Current care pathways for patients suffering from idiopathic SSNHL appear to vary considerably in terms of the type of service patients first present to, their subsequent

For peer review only

referral, length of time between onset of symptoms, presentation and start of treatment, the treatment plan as well as follow-up. The mainstay of treatment options for idiopathic SSNHL include oral steroids, intratympanic steroid injections or a combination of both.(5) NICE (National Institute for Health and Care Excellence), in their recent guidelines on hearing loss [NG98], highlighted the paucity of evidence assessing the comparative effectiveness of these treatments; and issued a high priority research recommendation to identify the most effective route of administration of steroids as a first-line treatment for idiopathic SSNHL.[10] The NIHR HTA (National Institute for Health Research, Health Technology Assessment) Programme has since released a commissioned call for a trial to address this question.[11]

For such a trial to be run effectively, reliable information is needed on patients with SSNHL: where they present, numbers, demographics, treatment pathways, as well as outcomes. This study will collect these data in a nationwide prospective cohort study of adult patients presenting with SSNHL across 97 NHS Trusts. The study will be delivered through INTEGRATE (National ENT Trainee Research Network), SFO-UK (Student and Foundation Doctors in Otolaryngology), NIHR Clinical Research Network (CRN) Audiology Champions, and the NIHR CRN. Importantly, this study will not only provide key data to inform trial design and delivery, but also a unique dataset to develop a prognostic model to predict recovery for patients with SSNHL.

Objectives

1. To map the patient pathway and identify the characteristics of adult patients presenting to NHS ENT and hearing services with SSNHL.

For peer review only

2. To develop a prognostic model to predict recovery for patients with idiopathic SSNHL.

3. Establish the impact of idiopathic SSNHL on patients' quality of life (QoL)

METHODS AND ANALYSIS

Study design

National multicentre prospective observational cohort study. The study will be reported in accordance with STROBE and 'Transparent Reporting of a multi variable prognostic model for Individual Prognosis or Diagnosis' (TRIPOD) reporting guidelines for observational studies.[12]

Setting

A multicentre study taking place across 97 NHS Trusts providing ENT and Hearing Services.

Inclusion criteria

- Adult patients (male or female) aged over 16 years of age presenting to NHS ENT and hearing services with SSNHL.

  AND

- Diagnosed with a hearing loss in one or both ears of 30 dB HL or more, over at least 3 contiguous frequencies, between 250, 500, 1000, 2000, 4000 and 8000 Hz.

  AND

- Willing and able to provide written informed consent.

Exclusion criteria

- Patients with mixed or conductive hearing loss (CHL). CHL will be defined as a 'true' air-bone gap of 15 dB HL or more in 3 or more contiguous frequencies between 500, 1000, 2000, 4000 Hz.

Sample size and recruitment

The minimum sample size for developing a multivariable prognostic model was estimated using the method reported by Riley et al.[12,13] Assuming a prevalence (i.e. full recovery of SSNHL) of between 32% and 65%,[2,8,9] a Nagelkerke's R-squared of 0.5,[13] a 0.05 margin of error in estimation of intercept, and a shrinkage factor of ≥ 0.95, with our prespecified number of predictor parameters of 15, resulted in a minimum sample size of 510 patients.

Ninety-seven NHS Trusts across the United Kingdom have been identified as study sites via INTEGRATE, SFO-UK, NIHR CRN Audiology Champions, and the NIHR CRN.

Candidate predictors for prognostic model

Age; gender; social class, presence of vestibular symptoms; precipitating illness; pattern of hearing loss; severity of hearing loss; time between onset of symptoms and treatment; treatment(s) received.

Pattern of hearing loss will be defined as: low frequency loss (250, 500 Hz), mid frequency loss (1000, 2000 Hz), high frequency loss (4000 Hz and 8000 Hz), flat (all frequencies), sloping (progressive loss across frequencies). Severity of hearing loss (on presentation) will be defined as follows: Pure tone average (PTA) across 6 frequencies (250, 500, 1000, 2000, 4000 and 8000 Hz), classified as: mild (25-40 dB loss), moderate (41-70 dB loss), severe (71-

For peer review only

95 dB loss) and profound (>95 dB loss).

Primary outcome:

The change in auditory function in the affected ear from initial presentation to follow-up (at any one time between 6 and 16 weeks from onset of symptoms). Auditory function will be defined as the PTA of air conduction thresholds at 250, 500, 1000, 2000, 4000 and 8000 Hz. If multiple pure tone audiograms have been carried out between 6 and 16 weeks, the most recent pure tone audiogram will be used for the calculation of auditory function.

Change in auditory function classified as:

1) Full recovery: Final PTA in affected ear within 10 dB of PTA of unaffected ear (≤ 10dB)
2) Partial to no recovery: Final PTA in affected ear ≥ 10dB of PTA of unaffected ear.

Secondary outcomes:

1) Degree of change in auditory function:

- Complete recovery: Final PTA in affected ear within 10 dB of PTA of unaffected ear (≤10dB)
- Marked recovery: PTA improvement ≥30 dB (and final PTA in affected ear ≥ 10dB of PTA of unaffected ear)
- Slight recovery: PTA improvement ≥10dB and <30 dB (and final PTA in affected ear ≥10 dB of PTA of unaffected ear)
- No improvement: PTA improvement <10 dB (and final PTA in affected ear ≥10 dB of PTA of unaffected ear)

For peer review only

2) Change in QoL score from initial presentation to follow-up at any one time between 6 and 16 weeks following treatment. QoL will be measured with the Hearing Handicap Inventory for Adults (HHIA) (for patients under 65 years of age) or Hearing Handicap Inventory for Elderly (HHIE) (for patients over 65 years of age) and the Health Utility Index Mark 3 (HUI3). QoL data will be collected from a subset of 20 Trusts.

Statistical analysis

We will develop a multivariable prognostic model to predict recovery for patients with SSNHL presenting to NHS ENT and Hearing services. Analyses will be conducted on the candidate predictors specified above (n=15). Missing outcome data at the end of the study will be imputed using multiple imputation by a chained equations procedure.[14] Internal validation will be performed to quantify optimism in the predictive performance (calibration and discrimination) of the developed model using bootstrapping techniques. Bootstrapping techniques provide information on the performance of the model in comparable datasets and generate a shrinkage factor to adjust the regression coefficients.[15,16] Statistical analysis will be carried out using R (version 3.6.2; R Foundation for Statistical Computing, Vienna, Austria).

The mean change in HHIA, HHIE and HUI3 scores across patients will be calculated from initial presentation to follow up (any one time between 6 and 16 weeks). We will use the non-parametric Wilcoxon and McNemar–Bowker tests with a significance level of 5%. Statistical data analysis will be carried out using the SPSS program 19.0 (SPSS, Chicago, IL, USA).

For peer review only

Study procedures

A summary of study procedures is outlined in Figure 1. Table 1 provides a summary of all data to be collected. Adult patients with SSNHL presenting to any of the 97 NHS trusts will be assessed for eligibility. In 20 of 97 NHS Trusts, all data identified in Table 1 will be collected. In the remaining 77 Trusts, all data except for QoL data will be collected. In centres where no QoL data are collected, there will be no deviation from the usual standard of care for any patient, and routinely collected patient data will be anonymised; therefore, consent is not required. For centres where QoL data are collected, patients will be provided with a study information sheet and requested to give written consent to participate in the study.

GENERAL INFORMATION
Patient demographics and socioeconomic status
Past medical history and social history
PATIENT'S FIRST PRESENTATION TO ENT
Hospital and service patient presenting to
Date of presentation
Referral pathway and treatments received up until first presentation to ENT
Clinical history of SSNHL
Examination findings
Audiometry results
Imaging requests/results
Laboratory test requests/results
Hearing Handicap Inventory for Adults (HHIA) questionnaire score OR Hearing Handicap Inventory for Elderly (HHIE) questionnaire score*
Health Utility Index Mark 3 (HUI3) questionnaire score*
Treatment plan
Cause of SSNHL identified
Follow-up details
FOLLOW-UP (6-16 WEEKS AFTER ONSET OF SYMPTOMS)
Date of presentation
Examination findings
Audiometry results
Imaging requests/results
Laboratory test requests/results
Hearing Handicap Inventory for Adults (HHIA) questionnaire score OR Hearing Handicap Inventory for Elderly (HHIE) questionnaire score*
Health Utility Index Mark 3 (HUI3) questionnaire score*

For peer review only

Treatments provided since first visit to ENT
Cause of SSNHL identified
Adverse events

Table 1 Data to be collected *To be collected at Quality of Life (QoL) sites only (20 of 97 sites)

Patient follow-up data will be collected at 6 to 16 weeks from onset of symptoms. All data collected will be uploaded onto REDCap, a secure online data collection platform.[17,18] All data will be transferred and stored in accordance with data governance regulations (Data Protection Act 2018).

Patient and public involvement

Three patients suffering from SSNHL were involved in the design of this study from its inception. They were recruited from a database of patients interested in taking part in Patient and Public Involvement (PPI) activities. They reviewed and gave input on study objectives and design, including reviewing participant information sheets and consent forms to ensure clarity. This group will also be involved in monitoring study progress and in its dissemination including co-authoring a lay summary report.

ETHICS AND DISSEMINATION

Ethical approval:

Health Research Authority (HRA) and NHS Research Ethics Committee has approved the study. IRAS project ID: 258494; Protocol number: NCT04108598; REC reference: 19/NW/0556

Risks, Burdens and Benefits

For peer review only

There are no anticipated risks or direct benefits to participants.

Dissemination

Results will be presented at local and inter(national) meetings and published in the scientific and grey literature for the attention of professional and scientific audiences. Publication and presentation of the final results will be on behalf of all study sites and collaborators. A lay summary report will be published for patients and members of the public.

For peer review only

References

1. Hughes GB, Freedman MA, Haberkamp TJ, et al. Sudden sensorineural hearing loss. *Otolaryngol Clin North Am* 1996;29(3):393–405.

2. Chandrasekhar SS, Tsai Do BS, Schwartz SR, et al. Clinical practice guideline: sudden hearing loss (update). *Otolaryngol Head Neck Surg* 2019;161(1 Suppl).

3. Levy JM, Amedee RG. In reference to Systematic review of the evidence for the etiology of adult sudden sensorineural hearing loss. *Laryngoscope* 2010;120(11):2347.

4. NICE. Hearing loss. Hearing loss in adults: Assessment and Management. 2018.

5. Harkonen K, Kivekas I, Rautiainen M, et al. Quality of Life and Hearing Eight Years After Sudden Sensorineural Hearing Loss. *Laryngoscope* 2017;127(4):927–31.

6. Chen J, Liang J, Ou J, et al. Mental health in adults with sudden sensorineural hearing loss: an assessment of depressive symptoms and its correlates. *J Psychosom Res* 2013;75(1):72–4.

7. Sano H, Okamoto M, Ohhashi K, et al. Quality of life reported by patients with idiopathic sudden sensorineural hearing loss. *Otol Neurotol* 2013;34(1):36–40.

8. Mattox, DE, Simmons, FB. Natural history of sudden sensorineural hearing loss. *Ann Otol Rhinol Laryngol* 1977;86:463-480

9. Stachler RJ, Chandrasekhar SS, Archer SM, et al. Clinical practice guideline: sudden hearing loss. *Otolaryngol Head Neck Surg* 2012;146(3)(suppl):S1-S35.

10. National Institute for Health and Clinical Excellence. Hearing loss in adults: assessment and management NICE guideline [NG98], 2018. Available: https://www.nice.org.uk/guidance/ng98

11. National Institute for Health Research. 19/128 Administration routes of steroids in the first-line treatment of idiopathic sudden sensorineural hearing loss, 2019. Available: https://www.nihr.ac.uk/documents/19128-administration-routes-of-steroids-in-the-first-line-treatment-of-idiopathic-sudden-sensorineural-hearing-loss/22731

12. Collins GS, Reitsma JB, Altman DG, et al. Transparent Reporting of a multivariable prediction model for Individual Prognosis Or Diagnosis (TRIPOD): the TRIPOD Statement. *Br J Surg 2015*;102(3):148–58.

13. Riley RD, Snell KI, Ensor J, et al. Minimum sample size for developing a multivariable prediction model: PART II - binary and time-to-event outcomes. *Stat Med* 2019;38(7):1276-1296.

14. van Buuren, S. Flexible Imputation of Missing Data. 2nd Edition. Boca Raton, FL: Chapman and Hall/CRC; 2018

15. Harrell Jr, FE. Regression Modeling Strategies. With Applications to Linear Models, Logistic and Ordinal Regression, and Survival Analysis. Cham: Springer International Publishing Switzerland; 2015

16. Steyerberg, EW. Clinical Prediction Models. A Practical Approach to Development, Validation, and Updating. 2nd edition. Cham: Springer Nature Switzerland AG; 201920.

17. Harris PA, Taylor R, Thielke R, et al. Research electronic data capture (REDCap) – A metadata-driven methodology and workflow process for providing translational research informatics support, *J Biomed Inform*. 2009;42(2):377-81.

18. Harris PA, Taylor R, Minor BL, et al. The REDCap consortium: Building an international community of software partners, *J Biomed Inform*. 2019 May 9 [doi: 10.1016/j.jbi.2019.103208]

For peer review only

Author Statement:_ The SeaSHeL Academic team were involved in writing this protocol.

RM, YP, NM, TF, HB, MK, JM, GH, TC, MA and AS made substantial contributions to the conception and design of the work. RM, YP, NM, TF, HB, MK, JM, GH, TC, MA and AS were involved in drafting the article and revising it critically. RM, AK, AWC, CT, EM, PL and

AS approved the final version to be published. RM, YP, NM, TF, HB, MK, JM, GH, TC, MA and AS agree to be accountable for all aspects of the work in ensuring that questions related to the accuracy or integrity of any part of the work are appropriately investigated and resolved.

Funding:

RM, AS, HB, NM, JM, TF are supported by the NIHR UCLH BRC.

Sensorion provided a small grant towards administrative support

Conflicts of Interests

Sensorion is an inner ear disease company and Sponsor of the NIHR Clinical Research Network Portfolio study, Audible-S (CPMS 39560, IRAS 248645). Sensorion has not been involved in the design of this study, nor will have access to the study data, be involved in data analysis or write-up of results.

Data Statement:

Dataset yet to be published.

Word count: 1792

Acknowledgements: Nil

Legends:

Figure 1 Summary of Study Procedures

A brief outline of study procedures from patient recruitment to data analysis.

For peer review only

Figure 1: Summary of Study Procedures

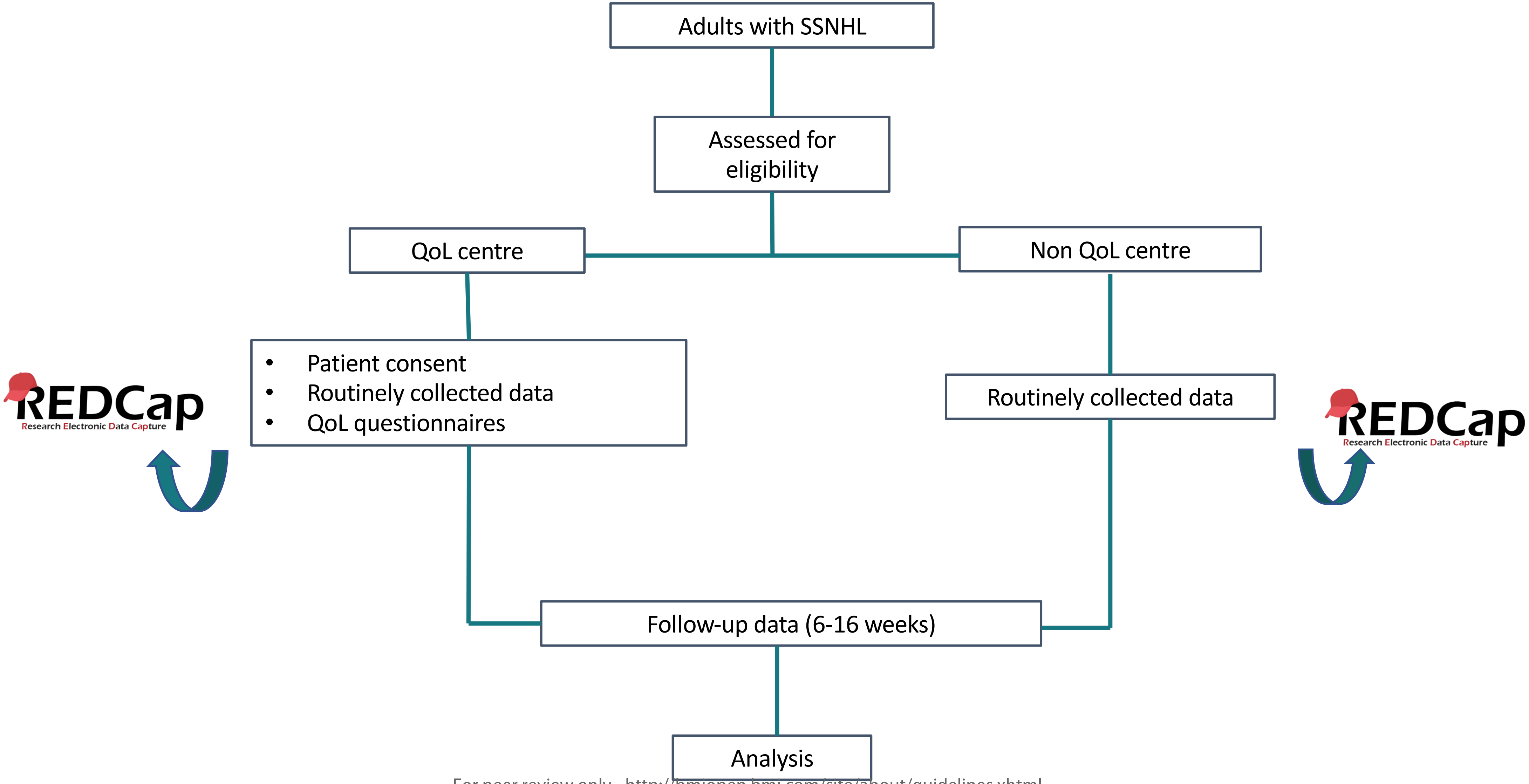

BMJ Open

Prognostic factors for outcomes of idiopathic Sudden Sensorineural Hearing Loss: protocol for the SeaSHeL national prospective cohort study

Journal:	*BMJ Open*
Manuscript ID	bmjopen-2020-038552.R1
Article Type:	Protocol
Date Submitted by the Author:	22-Jul-2020
Complete List of Authors:	Mandavia, Rishi; National Institute for Health Research, University College London Hospitals Biomedical Research Centre; University College London Ear Institute, evidENT<br>Hannink, Gerjon; Radboudumc, Department of Operating Rooms<br>Ahmed, Muhammad ; University College London Ear Institute, evidENT; University of Leeds School of Medicine<br>Premakumar, Yaami; Chelsea and Westminster Hospital<br>Chu, Timothy; 4. SFO UK Students and Foundation Doctors in Otolaryngology; Newcastle University School of Clinical Medical Sciences<br>Blackshaw, Helen; National Institute for Health Research, University College London Hospitals Biomedical Research Centre; University College London Ear Institute, evidENT<br>Ferdous, Tanjinah; National Institute for Health Research, University College London Hospitals Biomedical Research Centre; University College London Ear Institute, evidENT<br>Mehta, Nishchay; National Institute for Health Research, University College London Hospitals Biomedical Research Centre; University College London Ear Institute, evidENT<br>Manjaly, Joseph; National Institute for Health Research, University College London Hospitals Biomedical Research Centre; University College London Ear Institute, evidENT<br>Khan , Maha<br>Schilder, Anne; National Institute for Health Research, University College London Hospitals Biomedical Research Centre; University College London Ear Institute, evidENT
**Primary Subject Heading**:	Ear, nose and throat/otolaryngology
Secondary Subject Heading:	Epidemiology, Evidence based practice, Health informatics, Health services research
Keywords:	OTOLARYNGOLOGY, Adult otolaryngology < OTOLARYNGOLOGY, Audiology < OTOLARYNGOLOGY

I, the Submitting Author has the right to grant and does grant on behalf of all authors of the Work (as defined in the below author licence), an exclusive licence and/or a non-exclusive licence for contributions from authors who are: i) UK Crown employees; ii) where BMJ has agreed a CC-BY licence shall apply, and/or iii) in accordance with the terms applicable for US Federal Government officers or employees acting as part of their official duties; on a worldwide, perpetual, irrevocable, royalty-free basis to BMJ Publishing Group Ltd ("BMJ") its licensees and where the relevant Journal is co-owned by BMJ to the co-owners of the Journal, to publish the Work in this journal and any other BMJ products and to exploit all rights, as set out in our licence.

The Submitting Author accepts and understands that any supply made under these terms is made by BMJ to the Submitting Author unless you are acting as an employee on behalf of your employer or a postgraduate student of an affiliated institution which is paying any applicable article publishing charge ("APC") for Open Access articles. Where the Submitting Author wishes to make the Work available on an Open Access basis (and intends to pay the relevant APC), the terms of reuse of such Open Access shall be governed by a Creative Commons licence – details of these licences and which Creative Commons licence will apply to this Work are set out in our licence referred to above.

Other than as permitted in any relevant BMJ Author's Self Archiving Policies, I confirm this Work has not been accepted for publication elsewhere, is not being considered for publication elsewhere and does not duplicate material already published. I confirm all authors consent to publication of this Work and authorise the granting of this licence.

For peer review only

Prognostic factors for outcomes of idiopathic Sudden Sensorineural Hearing Loss: protocol for the SeaSHeL national prospective cohort study

Rishi Mandavia[1,2,3,4,5] (r.mandavia@ucl.ac.uk),

Gerjon Hannink[6] (gerjon.hannink@radboudumc.nl),

Muhammad N Ahmed[2,10] (um17mna@leeds.ac.uk),

Yaami Premakumar[7] (yaami.p@gmail.com),

Timothy Shun Man Chu[4,8] (t.chu1@newcastle.ac.uk),

Helen Blackshaw[1,2] (h.blackshaw@ucl.ac.uk),

Tanjinah Ferdous[1,2,5] (tanjinah.ferdous@nhs.net),

Nishchay Mehta[1,2,3,5] (nishchay.mehta.12@alumni.ucl.ac.uk),

Joseph Manjaly[1,2,5] (joseph.manjaly@nhs.net),

Maha Khan[3,4,9] (maha.khan@doctor.org.uk),

Anne G M Schilder[1,2,5] (a.schilder@ucl.ac.uk)

On behalf of the SeaSHeL Collaborative

1. National Institute for Health Research (NIHR), University College London Hospitals Biomedical Research Centre, 90 Tottenham Court Road, W1T 4TJ, United Kingdom
2. evidENT, Ear Institute, University College London, 90 Tottenham Court Road, W1T 4TJ, United Kingdom
3. INTEGRATE The National ENT Trainee Research Collaborative, United Kingdom
4. SFO UK Students and Foundation Doctors in Otolaryngology, United Kingdom
5. Royal National ENT and Eastman Dental Hospitals, University College London Hospitals Trust, London, United Kingdom

For peer review only

6. Radboud University Medical Centre, Nijmegen, Netherlands

7. Chelsea and Westminster Hospital, London, United Kingdom

8. Newcastle University Medical School, United Kingdom

9. Health Education North West, United Kingdom

10. University of Leeds School of Medicine, United Kingdom

Corresponding author.

Dr Rishi Mandavia

Address: NIHR UCLH BRC and evidENT, Ear Institute, University College London, 90 Tottenham Court Road, London W1T 4TJ

Email: r.mandavia@ucl.ac.uk

Telephone: +44 (0) 20 3108 9327

Word Count: 1792

Keywords

Sudden Sensorineural Hearing Loss, Prospective cohort study, Quality of life, Prognostic model, Steroids

For peer review only

ABSTRACT:

Introduction

The mainstay of treatment for idiopathic sudden sensorineural hearing loss (SSNHL) includes oral steroids, intratympanic steroid injections or a combination of both. NICE, in their recent hearing loss guidelines, highlighted the paucity of evidence assessing the comparative effectiveness of these treatments; and the NIHR HTA Programme has since released a commissioned call for a trial to identify the most effective route of administration of steroids as a first-line treatment for idiopathic SSNHL. For such trials to be run effectively, reliable information is needed on patients with SSNHL: where they present, numbers, demographics, treatment pathways, as well as outcomes. This study will collect these data in a nationwide cohort study of patients presenting with SSNHL across 97 NHS Trusts. The study will be delivered through ENT trainee networks, the NIHR Clinical Research Network (CRN) Audiology Champions, and the NIHR CRN. Importantly, this study will also provide a dataset to develop a prognostic model to predict recovery for patients with idiopathic SSNHL.

Objectives:

1. To map the patient pathway and identify the characteristics of adult patients presenting to NHS ENT and hearing services with SSNHL.

2. To develop a prognostic model to predict recovery for patients with idiopathic SSNHL.

3. To establish the impact of idiopathic SSNHL on patients' quality of life (QoL)

Methods and analysis

-Study design: National multicentre prospective cohort study across 97 NHS Trusts.

For peer review only

-Inclusion criteria: Adult patients presenting to NHS ENT and hearing services with SSNHL.

-Outcomes: Change in auditory function; change in QoL score.

-Analysis: Multivariable prognostic model, using pre-specified candidate predictors. Mean change in QoL scores will be calculated from initial presentation to follow up.

Ethics and dissemination

Health Research Authority and NHS Research Ethics Committee (REC) approved the study. Publication will be on behalf of all study sites and collaborators.

Registration details

Clinicaltrials.gov NCT04108598

ARTICLE SUMMARY

Strengths and limitations of this study

- Taking place across 97 NHS Trusts, the SeaSHeL study represents the first nationwide prospective cohort study of adults presenting to ENT and hearing services with SSNHL.
- It is uniquely delivered by INTEGRATE (National ENT Trainee Research Network), SFO-UK (Student and Foundation Doctors in Otolaryngology), NIHR Clinical Research Network (CRN) Audiology Champions, and the NIHR CRN.
- We acknowledge that duration between onset of SSNHL and presentation to ENT services as well as follow-up times and treatment strategies will vary across centres and cases, which may impact measured outcomes; however identifying differences in the patient pathway will form an important study finding.

For peer review only

INTRODUCTION

Each year approximately 20 people per 100,000 experience a sudden hearing loss in one ear that is located in the inner ear.[1] This is called sudden sensorineural hearing loss, SSNHL, defined as a rapid loss of hearing occurring over three days, of 30 dB (decibel) or more, over at least 3 contiguous sound frequencies.[2] SSNHL is predominantly unilateral and the degree of hearing loss can range from mild to profound. In 71% to 90% of cases, the cause is unknown despite investigation, and these cases are termed idiopathic SSNHL.[3,4]

Idiopathic SSNHL is a serious condition, adversely impacting people's lives[5] with research showing associations with emotional distress, depression, difficulties at work and impaired social integration.[6,7] Its prognosis is poorly understood and may depend on age, comorbidities, degree of hearing loss, audiometric configuration, presence of vertigo, treatment received, time between onset of hearing loss and treatment and other factors. It is estimated that 32% to 65% of cases of idiopathic SSNHL recover spontaneously, although clinical experience suggests that this may be an overestimation, with further research required in this area.[2,8,9]

Current care pathways for patients suffering from SSNHL appear to vary considerably in terms of the type of service patients first present to, their subsequent referral, length of time between onset of symptoms, presentation and start of treatment, the treatment plan as well as follow-up. The mainstay of treatment options for idiopathic SSNHL include oral steroids, intratympanic steroid injections or a combination of both.(5) NICE (National

For peer review only

Institute for Health and Care Excellence), in their recent guidelines on hearing loss [NG98], highlighted the paucity of evidence assessing the comparative effectiveness of these treatments; and issued a high priority research recommendation to identify the most effective route of administration of steroids as a first-line treatment for idiopathic SSNHL.[10] The NIHR HTA (National Institute for Health Research, Health Technology Assessment) Programme has since released a commissioned call for a trial to address this question.[11]

For such a trial to be run effectively, reliable information is needed on patients with SSNHL: where they present, numbers, demographics, treatment pathways, as well as outcomes. This study will collect these data in a nationwide prospective cohort study of adult patients presenting with SSNHL across 97 NHS Trusts. The study will be delivered through INTEGRATE (National ENT Trainee Research Network), SFO-UK (Student and Foundation Doctors in Otolaryngology), NIHR Clinical Research Network (CRN) Audiology Champions, and the NIHR CRN. Importantly, this study will not only provide key data to inform trial design and delivery, but also a unique dataset to develop a prognostic model to predict recovery for patients with idiopathic SSNHL.

For peer review only

Objectives

1. To map the patient pathway and identify the characteristics of adult patients presenting to NHS ENT and hearing services with SSNHL.

2. To develop a prognostic model to predict recovery for patients with idiopathic SSNHL.

3. To establish the impact of idiopathic SSNHL on patients' quality of life (QoL)

METHODS AND ANALYSIS

Study design

National multicentre prospective observational cohort study. The study will be reported in accordance with STROBE and 'Transparent Reporting of a multi variable prognostic model for Individual Prognosis or Diagnosis' (TRIPOD) reporting guidelines for observational studies.[12]

Setting

A multicentre study taking place across 97 NHS Trusts providing ENT and Hearing Services.

Inclusion criteria

- Adult patients (male or female) aged over 16 years of age presenting to NHS ENT and hearing services with SSNHL.

  AND
- Diagnosed with a hearing loss in one or both ears of 30 dB HL (absolute threshold) or more, over at least 3 contiguous frequencies, between the following specific frequencies: 250, 500, 1000, 2000, 4000 and 8000 Hz.

  AND
- Willing and able to provide written informed consent.

Exclusion criteria

- Patients with mixed or conductive hearing loss (CHL) in ear(s) with the sudden hearing loss. CHL will be defined as a 'true' air-bone gap of 15 dB or more in 3 or more

For peer review only

contiguous frequencies, between the following specific frequencies: 500, 1000, 2000, 4000 Hz.

Sample size and recruitment

The minimum sample size for developing a multivariable prognostic model to predict recovery for patients with idiopathic SSNHL was estimated using the method reported by Riley et al.[13] Assuming a prevalence (i.e. full recovery of idiopathic SSNHL) of between 32% and 65%,[2,8,9] a Nagelkerke's R-squared of 0.5,[13] a 0.05 margin of error in estimation of intercept, and a shrinkage factor of ≥ 0.95, with our prespecified number of predictor parameters of 15, resulted in a minimum sample size of 510 patients. Assuming that 70% of patients with SSNHL are idiopathic cases, this gives a total study minimal sample size of 730 patients.[3,4]

Ninety-seven NHS Trusts across the United Kingdom have been identified as study sites via INTEGRATE, SFO-UK, NIHR CRN Audiology Champions, and the NIHR CRN. An audit carried out in preparation of this study at 5 NHS trusts, over 6 months, revealed an average presentation rate of 1 patient with idiopathic SSNHL per trust per month. It appears therefore that our recruitment target over the study duration (32 months) is feasible.

Candidate predictors for prognostic model

Age; gender; social class, presence of vestibular symptoms; precipitating illness; pattern of hearing loss; severity of hearing loss; time between onset of symptoms and treatment; treatment(s) received.

For peer review only

Pattern of hearing loss will be defined as: low frequency loss (250, 500, 1000 Hz), mid frequency loss (500, 1000, 2000 Hz), high frequency loss (2000, 4000 Hz and 8000 Hz) and all frequencies). Severity of hearing loss (on presentation) will be defined as follows: Pure tone average (PTA) across 6 frequencies (250, 500, 1000, 2000, 4000 and 8000 Hz), classified as: mild (25-40 dB loss), moderate (41-70 dB loss), severe (71-95 dB loss) and profound (>95 dB loss)

Primary outcome:

The change in auditory function in the affected ear from initial presentation to follow-up (at any one time between 6 and 16 weeks from onset of symptoms). Auditory function will be defined as the PTA of air conduction thresholds between the following specific frequencies: 250, 500, 1000, 2000, 4000 and 8000 Hz. If multiple pure tone audiograms have been carried out between 6 and 16 weeks, the most recent pure tone audiogram will be used for the calculation of auditory function.

Change in auditory function classified as:

1) Full recovery:
   - Final PTA in affected ear within 10 dB (≤10dB) of PTA of baseline pure tone audiogram.

     OR, should a baseline pure tone audiogram not be available:
   - Final PTA in affected ear within 10 dB of PTA of unaffected ear.

     OR should both ears be affected with hearing loss:
   - Final PTA in affected ear within 10 dB of a normative reference (ISO7029).[14]

For peer review only

2) Partial to no recovery:

- Final PTA in affected ear ≥10dB of PTA of baseline pure tone audiogram.

  OR, should a baseline pure tone audiogram not be available:

- Final PTA in affected ear ≥10dB of PTA of unaffected ear.

  OR should both ears be affected with hearing loss:

- Final PTA in affected ear ≥10dB of PTA of a normative reference (ISO7029).[14]

For peer review only

Secondary outcomes:

1) Degree of change in auditory function:

- Complete recovery:

  - Final PTA in affected ear within 10 dB (≤10dB) of PTA of baseline pure tone audiogram

    OR should a baseline pure tone audiogram not be available:

  - Final PTA in affected ear within 10 dB of PTA of unaffected ear (≤ 10dB)

    OR should both ears be affected with hearing loss:

  - Final PTA in affected ear within 10 dB of a normative reference (ISO7029).[14]

- Marked recovery:

  - PTA improvement ≥30 dB (and final PTA in affected ear ≥10dB of PTA of baseline pure tone audiogram

    OR should a baseline pure tone audiogram not be available:

  - PTA improvement ≥30 dB (and final PTA in affected ear ≥10dB of PTA of unaffected ear)

    OR should both ears be affected with hearing loss:

- PTA improvement ≥30 dB (and final PTA in affected ear ≥10dB of a normative reference (ISO7029).[14]

- Slight recovery:
  - PTA improvement ≥10dB and <30 dB (and final PTA in affected ear ≥10dB of PTA of baseline pure tone audiogram

    OR should a baseline pure tone audiogram not be available:
  - PTA improvement ≥10dB and <30 dB (and final PTA in affected ear ≥10dB of PTA of unaffected ear)

    OR should both ears be affected with hearing loss:
  - PTA improvement ≥10dB and <30 dB (and final PTA in affected ear ≥10dB of a normative reference (ISO7029).[14]
- No improvement:
  - PTA improvement <10 dB (and final PTA in affected ear ≥10dB of PTA of baseline pure tone audiogram

    OR should a baseline pure tone audiogram not be available:
  - PTA improvement <10 dB (and final PTA in affected ear ≥10dB of PTA of unaffected ear)

    OR should both ears be affected with hearing loss:
  - PTA improvement of <10 dB (and final PTA in affected ear ≥10dB of a normative reference (ISO7029).[14]

2) Change in QoL score from initial presentation to follow-up at any one time between 6 and 16 weeks following treatment. QoL will be measured with the Hearing Handicap Inventory for Adults (HHIA) (for patients under 65 years of age) or Hearing Handicap

For peer review only

Inventory for Elderly (HHIE) (for patients over 65 years of age) and the Health Utility Index Mark 3 (HUI3). QoL data will be collected from a subset of 20 Trusts.

Out of limit thresholds obtained on the pure tone audiogram will be classified as a 96 dB loss at that specific frequency.

Statistical analysis

We will develop a prognostic model to predict recovery for patients with idiopathic SSNHL; and assess the impact of idiopathic SSNHL on patients' quality of life (QoL). A multivariable prognostic model will be developed using the candidate predictors specified above (n=15). Missing outcome data at the end of the study will be imputed using multiple imputation by a chained equations procedure.[15] Internal validation will be performed to quantify optimism in the predictive performance (calibration and discrimination) of the developed model using bootstrapping techniques. Bootstrapping techniques provide information on the performance of the model in comparable datasets and generate a shrinkage factor to adjust the regression coefficients.[16,17]

The mean change in HHIA, HHIE and HUI3 scores across patients with idiopathic SSNHL will be calculated from initial presentation to follow up (any one time between 6 and 16 weeks). We will use the non-parametric Wilcoxon and McNemar–Bowker tests with a significance level of 5%.-Statistical analysis will be carried out using R (version 3.6.2; R Foundation for Statistical Computing, Vienna, Austria).

Study procedures

For peer review only

A summary of study procedures is outlined in Figure 1. Table 1 provides a summary of all data to be collected. Adult patients with SSNHL presenting to any of the 97 NHS trusts will be assessed for eligibility. In 20 of 97 NHS Trusts, all data identified in Table 1 will be collected. In the remaining 77 Trusts, all data except for QoL data will be collected. In centres where no QoL data are collected, there will be no deviation from the usual standard of care for any patient, and routinely collected patient data will be anonymised; therefore, consent is not required. For centres where QoL data are collected, patients will be provided with a study information sheet and requested to give written consent to participate in the study.

GENERAL INFORMATION
Patient demographics and socioeconomic status
Past medical history and social history
PATIENT'S FIRST PRESENTATION TO ENT
Hospital and service patient presenting to
Date of presentation
Referral pathway and treatments received up until first presentation to ENT
Clinical history of SSNHL
Examination findings
Audiometry results
Imaging requests/results
Laboratory test requests/results
Vestibular function tests/results
Hearing Handicap Inventory for Adults (HHIA) questionnaire score OR Hearing Handicap Inventory for Elderly (HHIE) questionnaire score*
Health Utility Index Mark 3 (HUI3) questionnaire score*
Treatment plan
Cause of SSNHL identified
Follow-up details
FOLLOW-UP (6-16 WEEKS AFTER ONSET OF SYMPTOMS)
Date of presentation
Examination findings
Audiometry results
Imaging requests/results
Laboratory test requests/results
Hearing Handicap Inventory for Adults (HHIA) questionnaire score OR Hearing Handicap Inventory for Elderly (HHIE) questionnaire score*
Health Utility Index Mark 3 (HUI3) questionnaire score*
Treatments provided since first visit to ENT

For peer review only

Cause of SSNHL identified	
Adverse events	

Table 1 Data to be collected *To be collected at Quality of Life (QoL) sites only (20 of 97 sites)

Patient follow-up data will be collected at 6 to 16 weeks from onset of symptoms. All data collected will be uploaded onto REDCap, a secure online data collection platform.[18,19] All data will be transferred and stored in accordance with data governance regulations (Data Protection Act 2018).

For peer review only

Patient and public involvement

Three patients suffering from SSNHL were involved in the design of this study from its inception. They were recruited from a database of patients interested in taking part in Patient and Public Involvement (PPI) activities. They reviewed and gave input on study objectives and design, including reviewing participant information sheets and consent forms to ensure clarity. This group will also be involved in monitoring study progress and in its dissemination including co-authoring a lay summary report.

ETHICS AND DISSEMINATION

Ethical approval:

Health Research Authority (HRA) and NHS Research Ethics Committee has approved the study. IRAS project ID: 258494; Protocol number: NCT04108598; REC reference: 19/NW/0556

Risks, Burdens and Benefits

There are no anticipated risks or direct benefits to participants.

Dissemination

Results will be presented at local and inter(national) meetings and published in the scientific and grey literature for the attention of professional and scientific audiences. Publication and presentation of the final results will be on behalf of all study sites and collaborators. A lay summary report will be published for patients and members of the public.

Study start and end date

Start date: 7th October 2019

Planned end date: 1st May 2022

References

1. Hughes GB, Freedman MA, Haberkamp TJ, et al. Sudden sensorineural hearing loss. *Otolaryngol Clin North Am* 1996;29(3):393–405.

2. Chandrasekhar SS, Tsai Do BS, Schwartz SR, et al. Clinical practice guideline: sudden hearing loss (update). *Otolaryngol Head Neck Surg* 2019;161(1 Suppl).

3. Levy JM, Amedee RG. In reference to Systematic review of the evidence for the etiology of adult sudden sensorineural hearing loss. *Laryngoscope* 2010;120(11):2347.

4. NICE. Hearing loss. Hearing loss in adults: Assessment and Management. 2018.

5. Harkonen K, Kivekas I, Rautiainen M, et al. Quality of Life and Hearing Eight Years After Sudden Sensorineural Hearing Loss. *Laryngoscope* 2017;127(4):927–31.

6. Chen J, Liang J, Ou J, et al. Mental health in adults with sudden sensorineural hearing loss: an assessment of depressive symptoms and its correlates. *J Psychosom Res* 2013;75(1):72–4.

For peer review only

7. Sano H, Okamoto M, Ohhashi K, et al. Quality of life reported by patients with idiopathic sudden sensorineural hearing loss. *Otol Neurotol* 2013;34(1):36–40.

8. Mattox, DE, Simmons, FB. Natural history of sudden sensorineural hearing loss. *Ann Otol Rhinol Laryngol* 1977;86:463-480

9. Stachler RJ, Chandrasekhar SS, Archer SM, et al. Clinical practice guideline: sudden hearing loss. *Otolaryngol Head Neck Surg* 2012;146(3)(suppl):S1-S35.

10. National Institute for Health and Clinical Excellence. Hearing loss in adults: assessment and management NICE guideline [NG98], 2018. Available: https://www.nice.org.uk/guidance/ng98

11. National Institute for Health Research. 19/128 Administration routes of steroids in the first-line treatment of idiopathic sudden sensorineural hearing loss, 2019. Available: https://www.nihr.ac.uk/documents/19128-administration-routes-of-steroids-in-the-first-line-treatment-of-idiopathic-sudden-sensorineural-hearing-loss/22731

12. Collins GS, Reitsma JB, Altman DG, et al. Transparent Reporting of a multivariable prediction model for Individual Prognosis Or Diagnosis (TRIPOD): the TRIPOD Statement. *Br J Surg 2015*;102(3):148–58.

13. Riley RD, Snell KI, Ensor J, et al. Minimum sample size for developing a multivariable prediction model: PART II - binary and time-to-event outcomes. *Stat Med* 2019;38(7):1276-1296.

14. ISO 7029:2017 Acoustics – Statistical distribution of hearing thresholds related to age and gender. Available: https://www.iso.org/obp/ui/es/#iso:std:iso:7029:ed-3:v1:en

15. van Buuren, S. Flexible Imputation of Missing Data. 2nd Edition. Boca Raton, FL: Chapman and Hall/CRC; 2018

16. Harrell Jr, FE. Regression Modeling Strategies. With Applications to Linear Models, Logistic and Ordinal Regression, and Survival Analysis. Cham: Springer International Publishing Switzerland; 2015

17. Steyerberg, EW. Clinical Prediction Models. A Practical Approach to Development, Validation, and Updating. 2nd edition. Cham: Springer Nature Switzerland AG; 201920.

18. Harris PA, Taylor R, Thielke R, et al. Research electronic data capture (REDCap) – A metadata-driven methodology and workflow process for providing translational research informatics support, *J Biomed Inform*. 2009;42(2):377-81.

19. Harris PA, Taylor R, Minor BL, et al. The REDCap consortium: Building an

For peer review only

international community of software partners, *J Biomed Inform*. 2019 May 9

Author Statement:_ The SeaSHeL Academic team were involved in writing this protocol. RM, YP, NM, TF, HB, MK, JM, GH, TC, MA and AS made substantial contributions to the conception and design of the work. RM, YP, NM, TF, HB, MK, JM, GH, TC, MA and AS were involved in drafting the article and revising it critically. RM, AK, AWC, CT, EM, PL and AS approved the final version to be published. RM, YP, NM, TF, HB, MK, JM, GH, TC, MA and AS agree to be accountable for all aspects of the work in ensuring that questions related to the accuracy or integrity of any part of the work are appropriately investigated and resolved.

Funding:

RM, AS, HB, NM, JM, TF are supported by the NIHR UCLH BRC (Award number 254)

Sensorion provided a small grant towards administrative support (Grant number S6836)

Conflicts of Interests

Sensorion is an inner ear disease company and Sponsor of the NIHR Clinical Research Network Portfolio study, Audible-S (CPMS 39560, IRAS 248645). Sensorion has not been involved in the design of this study, nor will have access to the study data, be involved in data analysis or write-up of results.

Data Statement:

Dataset yet to be published.

For peer review only

Word count: 2242 words

Acknowledgements: Nil

Legends:

Figure 1 Summary of Study Procedures

A brief outline of study procedures from patient recruitment to data analysis.

For peer review only

Figure 1: Summary of Study Procedures

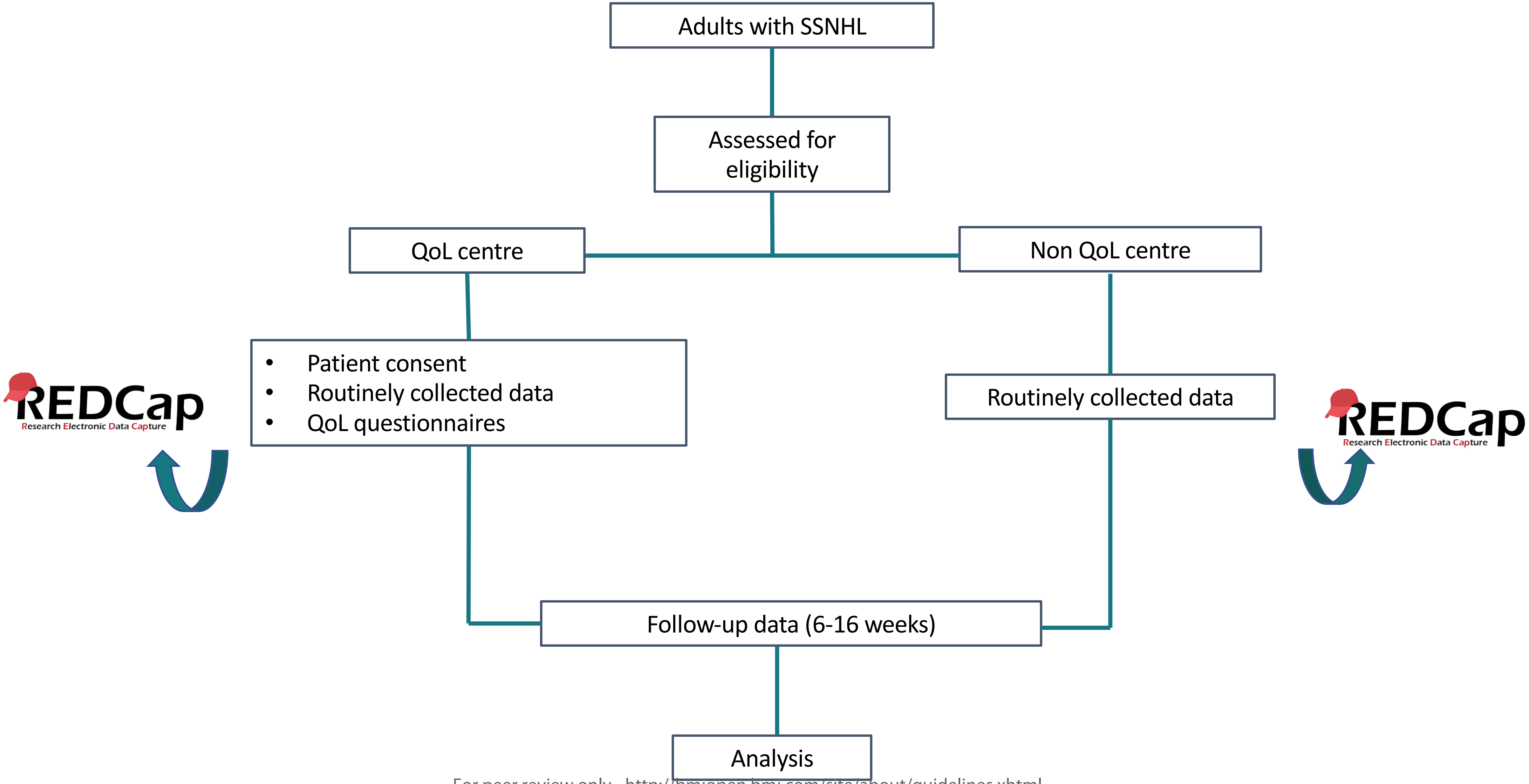

BMJ Open

Prognostic factors for outcomes of idiopathic Sudden Sensorineural Hearing Loss: protocol for the SeaSHeL national prospective cohort study

Journal:	*BMJ Open*
Manuscript ID	bmjopen-2020-038552.R2
Article Type:	Protocol
Date Submitted by the Author:	06-Aug-2020
Complete List of Authors:	Mandavia, Rishi; National Institute for Health Research, University College London Hospitals Biomedical Research Centre; University College London Ear Institute, evidENT<br>Hannink, Gerjon; Radboudumc, Department of Operating Rooms<br>Ahmed, Muhammad ; University College London Ear Institute, evidENT; University of Leeds School of Medicine<br>Premakumar, Yaami; Chelsea and Westminster Hospital<br>Chu, Timothy; 4. SFO UK Students and Foundation Doctors in Otolaryngology; Newcastle University School of Clinical Medical Sciences<br>Blackshaw, Helen; National Institute for Health Research, University College London Hospitals Biomedical Research Centre; University College London Ear Institute, evidENT<br>Ferdous, Tanjinah; National Institute for Health Research, University College London Hospitals Biomedical Research Centre; University College London Ear Institute, evidENT<br>Mehta, Nishchay; National Institute for Health Research, University College London Hospitals Biomedical Research Centre; University College London Ear Institute, evidENT<br>Manjaly, Joseph; National Institute for Health Research, University College London Hospitals Biomedical Research Centre; University College London Ear Institute, evidENT<br>Khan , Maha<br>Schilder, Anne; National Institute for Health Research, University College London Hospitals Biomedical Research Centre; University College London Ear Institute, evidENT
**Primary Subject Heading**:	Ear, nose and throat/otolaryngology
Secondary Subject Heading:	Epidemiology, Evidence based practice, Health informatics, Health services research
Keywords:	OTOLARYNGOLOGY, Adult otolaryngology < OTOLARYNGOLOGY, Audiology < OTOLARYNGOLOGY

SCHOLARONE™
Manuscripts

I, the Submitting Author has the right to grant and does grant on behalf of all authors of the Work (as defined in the below author licence), an exclusive licence and/or a non-exclusive licence for contributions from authors who are: i) UK Crown employees; ii) where BMJ has agreed a CC-BY licence shall apply, and/or iii) in accordance with the terms applicable for US Federal Government officers or employees acting as part of their official duties; on a worldwide, perpetual, irrevocable, royalty-free basis to BMJ Publishing Group Ltd ("BMJ") its licensees and where the relevant Journal is co-owned by BMJ to the co-owners of the Journal, to publish the Work in this journal and any other BMJ products and to exploit all rights, as set out in our licence.

The Submitting Author accepts and understands that any supply made under these terms is made by BMJ to the Submitting Author unless you are acting as an employee on behalf of your employer or a postgraduate student of an affiliated institution which is paying any applicable article publishing charge ("APC") for Open Access articles. Where the Submitting Author wishes to make the Work available on an Open Access basis (and intends to pay the relevant APC), the terms of reuse of such Open Access shall be governed by a Creative Commons licence – details of these licences and which Creative Commons licence will apply to this Work are set out in our licence referred to above.

Other than as permitted in any relevant BMJ Author's Self Archiving Policies, I confirm this Work has not been accepted for publication elsewhere, is not being considered for publication elsewhere and does not duplicate material already published. I confirm all authors consent to publication of this Work and authorise the granting of this licence.

For peer review only

Prognostic factors for outcomes of idiopathic Sudden Sensorineural Hearing Loss: protocol for the SeaSHeL national prospective cohort study

Rishi Mandavia[1,2,3,4,5] (r.mandavia@ucl.ac.uk),

Gerjon Hannink[6] (gerjon.hannink@radboudumc.nl),

Muhammad N Ahmed[2,10] (um17mna@leeds.ac.uk),

Yaami Premakumar[7] (yaami.p@gmail.com),

Timothy Shun Man Chu[4,8] (t.chu1@newcastle.ac.uk),

Helen Blackshaw[1,2] (h.blackshaw@ucl.ac.uk),

Tanjinah Ferdous[1,2,5] (tanjinah.ferdous@nhs.net),

Nishchay Mehta[1,2,3,5] (nishchay.mehta.12@alumni.ucl.ac.uk),

Joseph Manjaly[1,2,5] (joseph.manjaly@nhs.net),

Maha Khan[3,4,9] (maha.khan@doctor.org.uk),

Anne G M Schilder[1,2,5] (a.schilder@ucl.ac.uk)

On behalf of the SeaSHeL Collaborative

1. National Institute for Health Research (NIHR), University College London Hospitals Biomedical Research Centre, 90 Tottenham Court Road, W1T 4TJ, United Kingdom
2. evidENT, Ear Institute, University College London, 90 Tottenham Court Road, W1T 4TJ, United Kingdom
3. INTEGRATE The National ENT Trainee Research Collaborative, United Kingdom
4. SFO UK Students and Foundation Doctors in Otolaryngology, United Kingdom
5. Royal National ENT and Eastman Dental Hospitals, University College London Hospitals Trust, London, United Kingdom
6. Radboud University Medical Centre, Nijmegen, Netherlands

For peer review only

7. Chelsea and Westminster Hospital, London, United Kingdom

8. Newcastle University Medical School, United Kingdom

9. Health Education North West, United Kingdom

10. University of Leeds School of Medicine, United Kingdom

Corresponding author.

Dr Rishi Mandavia

Address: NIHR UCLH BRC and evidENT, Ear Institute, University College London, 90 Tottenham Court Road, London W1T 4TJ

Email: r.mandavia@ucl.ac.uk

Telephone: +44 (0) 20 3108 9327

Word Count: 1792

Keywords

Sudden Sensorineural Hearing Loss, Prospective cohort study, Quality of life, Prognostic model, Steroids

For peer review only

ABSTRACT:

Introduction

The mainstay of treatment for idiopathic sudden sensorineural hearing loss (SSNHL) includes oral steroids, intratympanic steroid injections or a combination of both. NICE, in their recent hearing loss guidelines, highlighted the paucity of evidence assessing the comparative effectiveness of these treatments; and the NIHR HTA Programme has since released a commissioned call for a trial to identify the most effective route of administration of steroids as a first-line treatment for idiopathic SSNHL. For such trials to be run effectively, reliable information is needed on patients with SSNHL: where they present, numbers, demographics, treatment pathways, as well as outcomes. This study will collect these data in a nationwide cohort study of patients presenting with SSNHL across 97 NHS Trusts. The study will be delivered through ENT trainee networks, the NIHR Clinical Research Network (CRN) Audiology Champions, and the NIHR CRN. Importantly, this study will also provide a dataset to develop a prognostic model to predict recovery for patients with idiopathic SSNHL. The study objectives are to: 1) Map the patient pathway and identify the characteristics of adult patients presenting to NHS ENT and hearing services with SSNHL. 2) Develop a prognostic model to predict recovery for patients with idiopathic SSNHL. 3) Establish the impact of idiopathic SSNHL on patients' quality of life (QoL).

Methods and analysis

-Study design: National multicentre prospective cohort study across 97 NHS Trusts.

-Inclusion criteria: Adult patients presenting to NHS ENT and hearing services with SSNHL.

-Outcomes: Change in auditory function; change in QoL score.

For peer review only

-Analysis: Multivariable prognostic model, using pre-specified candidate predictors. Mean change in QoL scores will be calculated from initial presentation to follow up.

Ethics and dissemination

Health Research Authority and NHS Research Ethics Committee (REC) approved the study. Publication will be on behalf of study sites and collaborators.

Registration details

Clinicaltrials.gov NCT04108598

ARTICLE SUMMARY

Strengths and limitations of this study

- Taking place across 97 NHS Trusts, the SeaSHeL study represents the first nationwide prospective cohort study of adults presenting to ENT and hearing services with SSNHL.
- It is uniquely delivered by INTEGRATE (National ENT Trainee Research Network), SFO-UK (Student and Foundation Doctors in Otolaryngology), NIHR Clinical Research Network (CRN) Audiology Champions, and the NIHR CRN.
- We acknowledge that duration between onset of SSNHL and presentation to ENT services as well as follow-up times and treatment strategies will vary across centres and cases, which may impact measured outcomes; however identifying differences in the patient pathway will form an important study finding.

For peer review only

INTRODUCTION

Each year approximately 20 people per 100,000 experience a sudden hearing loss in one ear that is located in the inner ear.[1] This is called sudden sensorineural hearing loss, SSNHL, defined as a rapid loss of hearing occurring over three days, of 30 dB (decibel) or more, over at least 3 contiguous sound frequencies.[2] SSNHL is predominantly unilateral and the degree of hearing loss can range from mild to profound. In 71% to 90% of cases, the cause is unknown despite investigation, and these cases are termed idiopathic SSNHL.[3,4]

Idiopathic SSNHL is a serious condition, adversely impacting people's lives[5] with research showing associations with emotional distress, depression, difficulties at work and impaired social integration.[6,7] Its prognosis is poorly understood and may depend on age, comorbidities, degree of hearing loss, audiometric configuration, presence of vertigo, treatment received, time between onset of hearing loss and treatment and other factors. It is estimated that 32% to 65% of cases of idiopathic SSNHL recover spontaneously, although clinical experience suggests that this may be an overestimation, with further research required in this area.[2,8,9]

Current care pathways for patients suffering from SSNHL appear to vary considerably in terms of the type of service patients first present to, their subsequent referral, length of time between onset of symptoms, presentation and start of treatment, the treatment plan as well as follow-up. The mainstay of treatment options for idiopathic SSNHL include oral steroids, intratympanic steroid injections or a combination of both.(5) NICE (National Institute for Health and Care Excellence), in their recent guidelines on hearing loss [NG98],

For peer review only

highlighted the paucity of evidence assessing the comparative effectiveness of these treatments; and issued a high priority research recommendation to identify the most effective route of administration of steroids as a first-line treatment for idiopathic SSNHL.[10] The NIHR HTA (National Institute for Health Research, Health Technology Assessment) Programme has since released a commissioned call for a trial to address this question.[11]

For such a trial to be run effectively, reliable information is needed on patients with SSNHL: where they present, numbers, demographics, treatment pathways, as well as outcomes. This study will collect these data in a nationwide prospective cohort study of adult patients presenting with SSNHL across 97 NHS Trusts. The study will be delivered through INTEGRATE (National ENT Trainee Research Network), SFO-UK (Student and Foundation Doctors in Otolaryngology), NIHR Clinical Research Network (CRN) Audiology Champions, and the NIHR CRN. Importantly, this study will not only provide key data to inform trial design and delivery, but also a unique dataset to develop a prognostic model to predict recovery for patients with idiopathic SSNHL.

For peer review only

Objectives

1. To map the patient pathway and identify the characteristics of adult patients presenting to NHS ENT and hearing services with SSNHL.

2. To develop a prognostic model to predict recovery for patients with idiopathic SSNHL.

3. To establish the impact of idiopathic SSNHL on patients’ quality of life (QoL).

METHODS AND ANALYSIS

Study design

National multicentre prospective observational cohort study. The study will be reported in accordance with STROBE and 'Transparent Reporting of a multi variable prognostic model for Individual Prognosis or Diagnosis' (TRIPOD) reporting guidelines for observational studies.[12]

Setting

A multicentre study taking place across 97 NHS Trusts providing ENT and Hearing Services.

Inclusion criteria

- Adult patients (male or female) aged over 16 years of age presenting to NHS ENT and hearing services with a history of sudden hearing loss (within a 72-hour window) that is sensorineural in nature.

  AND

- There is a decrease in hearing of ≥30 dB affecting at least 3 consecutive frequencies (between the following specific frequencies: 250, 500, 1000, 2000, 4000 and 8000 Hz), using a premorbid pure tone audiogram as a baseline reference. OR should a baseline pure tone audiogram not be available, the unaffected ear can be used a baseline reference. Or should both ears be affected by hearing loss, a normative reference (ISO7029)[13] can be used as the baseline reference.

  AND

- Willing and able to provide written informed consent.

For peer review only

Exclusion criteria

- Patients with mixed or conductive hearing loss (CHL) in ear(s) with the sudden hearing loss. CHL will be defined as a 'true' air-bone gap of 15 dB or more in 3 or more contiguous frequencies, between the following specific frequencies: 500, 1000, 2000, 4000 Hz.

Sample size and recruitment

The minimum sample size for developing a multivariable prognostic model to predict recovery for patients with idiopathic SSNHL was estimated using the method reported by Riley et al.[14] Assuming a prevalence (i.e. full recovery of idiopathic SSNHL) of between 32% and 65%,[2,8,9] a Nagelkerke's R-squared of 0.5,[14] a 0.05 margin of error in estimation of intercept, and a shrinkage factor of ≥ 0.95, with our prespecified number of predictor parameters of 15, resulted in a minimum sample size of 510 patients. Assuming that 70% of patients with SSNHL are idiopathic cases, this gives a total study minimal sample size of 730 patients.[3,4]

Ninety-seven NHS Trusts across the United Kingdom have been identified as study sites via INTEGRATE, SFO-UK, NIHR CRN Audiology Champions, and the NIHR CRN. An audit carried out in preparation of this study at 5 NHS trusts, over 6 months, revealed an average presentation rate of 1 patient with idiopathic SSNHL per trust per month. It appears therefore that our recruitment target over the study duration (32 months) is feasible.

Candidate predictors for prognostic model

For peer review only

Age; gender; social class, presence of vestibular symptoms; precipitating illness; pattern of hearing loss; severity of hearing loss; time between onset of symptoms and treatment; treatment(s) received.

Pattern of hearing loss will be defined as: low frequency loss (250, 500, 1000 Hz), mid frequency loss (500, 1000, 2000 Hz), high frequency loss (2000, 4000 Hz and 8000 Hz) and all frequencies). Severity of hearing loss (on presentation) will be defined as follows: Pure tone average (PTA) across 6 frequencies (250, 500, 1000, 2000, 4000 and 8000 Hz), classified as: mild (25-40 dB loss), moderate (41-70 dB loss), severe (71-95 dB loss) and profound (>95 dB loss)

Primary outcome:

The change in auditory function in the affected ear from initial presentation to follow-up (at any one time between 6 and 16 weeks from onset of symptoms). Auditory function will be defined as the PTA of air conduction thresholds between the following specific frequencies: 250, 500, 1000, 2000, 4000 and 8000 Hz. If multiple pure tone audiograms have been carried out between 6 and 16 weeks, the most recent pure tone audiogram will be used for the calculation of auditory function.

Change in auditory function classified as:

1) Full recovery:
   - Final PTA in affected ear within 10 dB (≤10 dB) of PTA of baseline pure tone audiogram.

     OR, should a baseline pure tone audiogram not be available:

For peer review only

- Final PTA in affected ear within 10 dB of PTA of unaffected ear.

  OR should both ears be affected with hearing loss:

- Final PTA in affected ear within 10 dB of a normative reference (ISO7029).[13]

2) Partial to no recovery:

- Final PTA in affected ear ≥10 dB of PTA of baseline pure tone audiogram.

  OR, should a baseline pure tone audiogram not be available:

- Final PTA in affected ear ≥10 dB of PTA of unaffected ear.

  OR should both ears be affected with hearing loss:

- Final PTA in affected ear ≥10 dB of PTA of a normative reference (ISO7029).[13]

Secondary outcomes:

1) Degree of change in auditory function:

- Complete recovery:

  - Final PTA in affected ear within 10 dB (≤10 dB) of PTA of baseline pure tone audiogram

    OR should a baseline pure tone audiogram not be available:

  - Final PTA in affected ear within 10 dB of PTA of unaffected ear (≤ 10 dB)

    OR should both ears be affected with hearing loss:

  - Final PTA in affected ear within 10 dB of a normative reference (ISO7029).[13]

- Marked recovery:

  - PTA improvement ≥30 dB (and final PTA in affected ear ≥10 dB of PTA of baseline pure tone audiogram

For peer review only

OR should a baseline pure tone audiogram not be available:

- PTA improvement ≥30 dB (and final PTA in affected ear ≥10 dB of PTA of unaffected ear)

OR should both ears be affected with hearing loss:

- PTA improvement ≥30 dB (and final PTA in affected ear ≥10 dB of a normative reference (ISO7029).[13]

For peer review only

- Slight recovery:
  - PTA improvement ≥10 dB and <30 dB (and final PTA in affected ear ≥10 dB of PTA of baseline pure tone audiogram

    OR should a baseline pure tone audiogram not be available:
  - PTA improvement ≥10 dB and <30 dB (and final PTA in affected ear ≥10dB of PTA of unaffected ear)

    OR should both ears be affected with hearing loss:
  - PTA improvement ≥10 dB and <30 dB (and final PTA in affected ear ≥10 dB of a normative reference (ISO7029).[13]
- No improvement:
  - PTA improvement <10 dB (and final PTA in affected ear ≥10 dB of PTA of baseline pure tone audiogram

    OR should a baseline pure tone audiogram not be available:
  - PTA improvement <10 dB (and final PTA in affected ear ≥10 dB of PTA of unaffected ear)

    OR should both ears be affected with hearing loss:
  - PTA improvement of <10 dB (and final PTA in affected ear ≥10 dB of a normative reference (ISO7029).[13]

2) Change in QoL score from initial presentation to follow-up at any one time between 6 and 16 weeks following treatment. QoL will be measured with the Hearing Handicap Inventory for Adults (HHIA) (for patients under 65 years of age) or Hearing Handicap Inventory for Elderly (HHIE) (for patients over 65 years of age) and the Health Utility Index Mark 3 (HUI3). QoL data will be collected from a subset of 20 Trusts.

Out of limit thresholds obtained on the pure tone audiogram will be classified as a 96 dB loss at that specific frequency.

Statistical analysis

We will develop a prognostic model to predict recovery for patients with idiopathic SSNHL; and assess the impact of idiopathic SSNHL on patients' quality of life (QoL). A multivariable prognostic model will be developed using the candidate predictors specified above (n=15). Missing outcome data at the end of the study will be imputed using multiple imputation by a chained equations procedure.[15] Internal validation will be performed to quantify optimism in the predictive performance (calibration and discrimination) of the developed model using bootstrapping techniques. Bootstrapping techniques provide information on the performance of the model in comparable datasets and generate a shrinkage factor to adjust the regression coefficients.[16,17]

The mean change in HHIA, HHIE and HUI3 scores across patients with idiopathic SSNHL will be calculated from initial presentation to follow up (any one time between 6 and 16 weeks). We will use the non-parametric Wilcoxon and McNemar–Bowker tests with a significance

For peer review only

level of 5%.-Statistical analysis will be carried out using R (version 3.6.2; R Foundation for Statistical Computing, Vienna, Austria).

Study procedures

A summary of study procedures is outlined in Figure 1. Table 1 provides a summary of all data to be collected. Adult patients with SSNHL presenting to any of the 97 NHS trusts will be assessed for eligibility. In 20 of 97 NHS Trusts, all data identified in Table 1 will be collected. In the remaining 77 Trusts, all data except for QoL data will be collected. In centres where no QoL data are collected, there will be no deviation from the usual standard of care for any patient, and routinely collected patient data will be anonymised; therefore, consent is not required. For centres where QoL data are collected, patients will be provided with a study information sheet and requested to give written consent to participate in the study.

GENERAL INFORMATION
Patient demographics and socioeconomic status
Past medical history and social history
PATIENT'S FIRST PRESENTATION TO ENT
Hospital and service patient presenting to
Date of presentation
Referral pathway and treatments received up until first presentation to ENT
Clinical history of SSNHL
Examination findings
Audiometry results
Imaging requests/results
Laboratory test requests/results (data on any laboratory test requested for a patient with SSNHL to be collected)
Vestibular function tests/results (data on any vestibular function test requested for a patient with SSNHL to be collected)
Hearing Handicap Inventory for Adults (HHIA) questionnaire score OR Hearing Handicap Inventory for Elderly (HHIE) questionnaire score*
Health Utility Index Mark 3 (HUI3) questionnaire score*
Treatment plan
Cause of SSNHL identified
Follow-up details

For peer review only

FOLLOW-UP (6-16 WEEKS AFTER ONSET OF SYMPTOMS)
Date of presentation
Examination findings
Audiometry results
Imaging requests/results
Laboratory test results (data on any laboratory test requested for a patient with SSNHL to be collected)
Vestibular function results (data on any vestibular function test requested for a patient with SSNHL to be collected)
Hearing Handicap Inventory for Adults (HHIA) questionnaire score OR Hearing Handicap Inventory for Elderly (HHIE) questionnaire score*
Health Utility Index Mark 3 (HUI3) questionnaire score*
Treatments provided since first visit to ENT
Cause of SSNHL identified
Adverse events

Table 1 Data to be collected *To be collected at Quality of Life (QoL) sites only (20 of 97 sites)

Patient follow-up data will be collected at 6 to 16 weeks from onset of symptoms. All data collected will be uploaded onto REDCap, a secure online data collection platform.[18,19] All data will be transferred and stored in accordance with data governance regulations (Data Protection Act 2018).

Patient and public involvement

Three patients suffering from SSNHL were involved in the design of this study from its inception. They were recruited from a database of patients interested in taking part in Patient and Public Involvement (PPI) activities. They reviewed and gave input on study objectives and design, including reviewing participant information sheets and consent forms to ensure clarity. This group will also be involved in monitoring study progress and in its dissemination including co-authoring a lay summary report.

ETHICS AND DISSEMINATION

For peer review only

Ethical approval:

Health Research Authority (HRA) and NHS Research Ethics Committee has approved the study. IRAS project ID: 258494; Protocol number: NCT04108598; REC reference: 19/NW/0556

Risks, Burdens and Benefits

There are no anticipated risks or direct benefits to participants.

Dissemination

Results will be presented at local and inter(national) meetings and published in the scientific and grey literature for the attention of professional and scientific audiences. Publication and presentation of the final results will be on behalf of all study sites and collaborators. A lay summary report will be published for patients and members of the public.

Study start and end date

Start date: 7th October 2019

Planned end date: 1st May 2022

References

1. Hughes GB, Freedman MA, Haberkamp TJ, et al. Sudden sensorineural hearing loss. *Otolaryngol Clin North Am* 1996;29(3):393–405.

2. Chandrasekhar SS, Tsai Do BS, Schwartz SR, et al. Clinical practice guideline: sudden hearing loss (update). *Otolaryngol Head Neck Surg* 2019;161(1 Suppl).

3. Levy JM, Amedee RG. In reference to Systematic review of the evidence for the etiology of adult sudden sensorineural hearing loss. *Laryngoscope*

For peer review only

2010;120(11):2347.

4. NICE. Hearing loss. Hearing loss in adults: Assessment and Management. 2018.

5. Harkonen K, Kivekas I, Rautiainen M, et al. Quality of Life and Hearing Eight Years After Sudden Sensorineural Hearing Loss. *Laryngoscope* 2017;127(4):927–31.

6. Chen J, Liang J, Ou J, et al. Mental health in adults with sudden sensorineural hearing loss: an assessment of depressive symptoms and its correlates. *J Psychosom Res* 2013;75(1):72–4.

7. Sano H, Okamoto M, Ohhashi K, et al. Quality of life reported by patients with idiopathic sudden sensorineural hearing loss. *Otol Neurotol* 2013;34(1):36–40.

8. Mattox, DE, Simmons, FB. Natural history of sudden sensorineural hearing loss. *Ann Otol Rhinol Laryngol* 1977;86:463-480

9. Stachler RJ, Chandrasekhar SS, Archer SM, et al. Clinical practice guideline: sudden hearing loss. *Otolaryngol Head Neck Surg* 2012;146(3)(suppl):S1-S35.

10. National Institute for Health and Clinical Excellence. Hearing loss in adults: assessment and management NICE guideline [NG98], 2018. Available: https://www.nice.org.uk/guidance/ng98

11. National Institute for Health Research. 19/128 Administration routes of steroids in the first-line treatment of idiopathic sudden sensorineural hearing loss, 2019. Available: https://www.nihr.ac.uk/documents/19128-administration-routes-of-steroids-in-the-first-line-treatment-of-idiopathic-sudden-sensorineural-hearing-loss/22731

12. Collins GS, Reitsma JB, Altman DG, et al. Transparent Reporting of a multivariable prediction model for Individual Prognosis Or Diagnosis (TRIPOD): the TRIPOD Statement. *Br J Surg 2015*;102(3):148–58.

13. ISO 7029:2017 Acoustics – Statistical distribution of hearing thresholds related to age and gender. Available: https://www.iso.org/obp/ui/es/#iso:std:iso:7029:ed-3:v1:en

14. Riley RD, Snell KI, Ensor J, et al. Minimum sample size for developing a multivariable prediction model: PART II - binary and time-to-event outcomes. *Stat Med* 2019;38(7):1276-1296.

15. van Buuren, S. Flexible Imputation of Missing Data. 2nd Edition. Boca Raton, FL: Chapman and Hall/CRC; 2018

16. Harrell Jr, FE. Regression Modeling Strategies. With Applications to Linear

For peer review only

Models, Logistic and Ordinal Regression, and Survival Analysis. Cham: Springer International Publishing Switzerland; 2015

17. Steyerberg, EW. Clinical Prediction Models. A Practical Approach to Development, Validation, and Updating. 2nd edition. Cham: Springer Nature Switzerland AG; 201920.

18. Harris PA, Taylor R, Thielke R, et al. Research electronic data capture (REDCap) – A metadata-driven methodology and workflow process for providing translational research informatics support, *J Biomed Inform*. 2009;42(2):377-81.

19. Harris PA, Taylor R, Minor BL, et al. The REDCap consortium: Building an international community of software partners, *J Biomed Inform*. 2019 May 9

Author Statement: The SeaSHeL Academic team were involved in writing this protocol. RM, YP, NM, TF, HB, MK, JM, GH, TC, MA and AS made substantial contributions to the conception and design of the work. RM, YP, NM, TF, HB, MK, JM, GH, TC, MA and AS were involved in drafting the article and revising it critically. RM, YP, NM, TF, HB, MK, JM, GH, TC, MA and AS approved the final version to be published. RM, YP, NM, TF, HB, MK, JM, GH, TC, MA and AS agree to be accountable for all aspects of the work in ensuring that questions related to the accuracy or integrity of any part of the work are appropriately investigated and resolved.

Funding:

RM, AS, HB, NM, JM, TF are supported by the NIHR UCLH BRC (Award number 254)

Sensorion provided a small grant towards administrative support (Grant number S6836)

Conflicts of Interests

For peer review only

Sensorion is an inner ear disease company and Sponsor of the NIHR Clinical Research Network Portfolio study, Audible-S (CPMS 39560, IRAS 248645). Sensorion has not been involved in the design of this study, nor will have access to the study data, be involved in data analysis or write-up of results.

Data Statement:

Dataset yet to be published.

Word count: 2242 words

Acknowledgements: Nil

Legends:

Figure 1 Summary of Study Procedures

A brief outline of study procedures from patient recruitment to data analysis.

For peer review only

Figure 1: Summary of Study Procedures

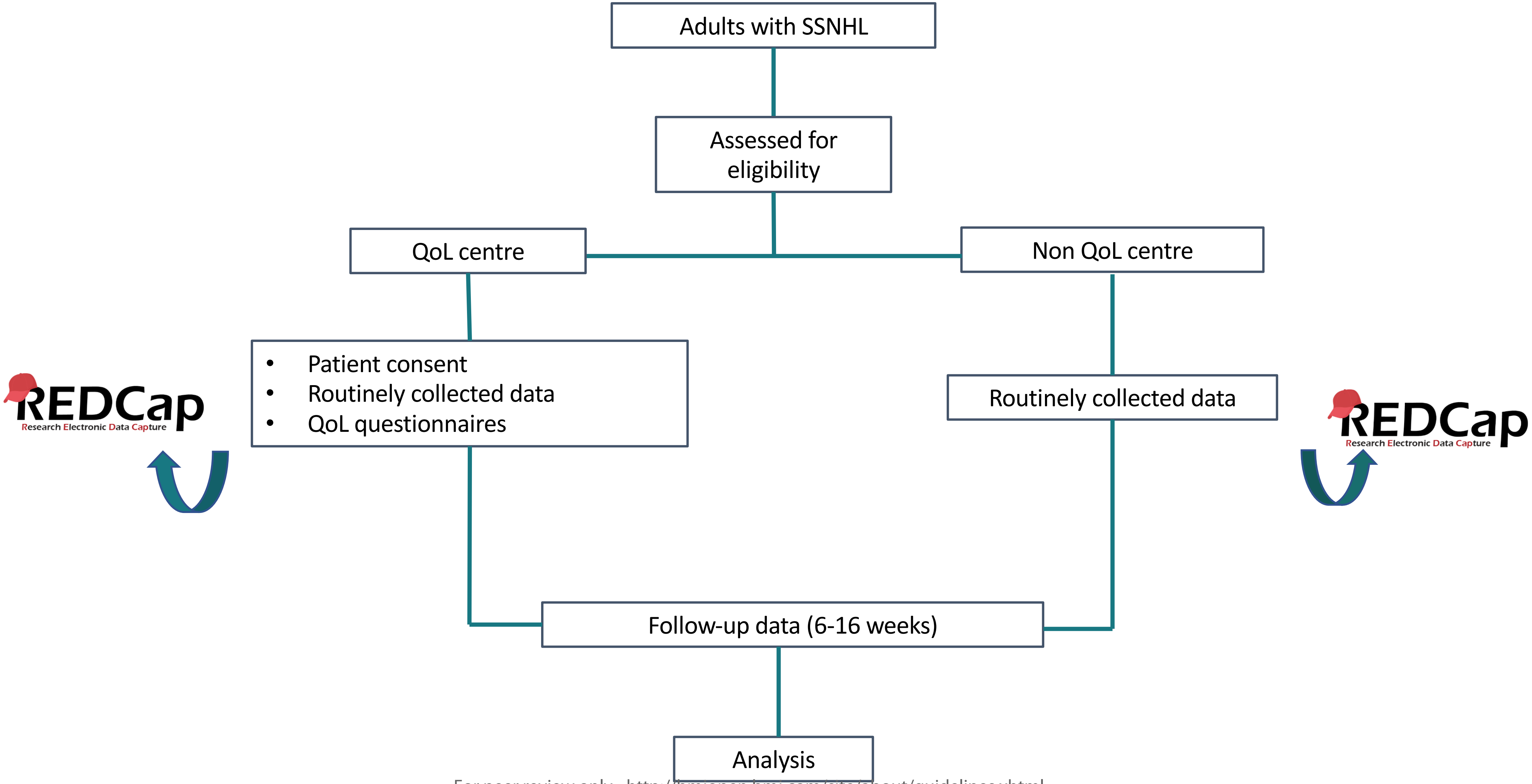

Supplement: Author's manuscript [file bmjopen-2020-038552.draft_revisions.pdf]
